# Variations in Human Herpesvirus Type 8 Seroprevalence in Native Americans, South America

**DOI:** 10.3201/eid1606.090961

**Published:** 2010-06

**Authors:** Vanda A.U.F. Souza, Francisco M. Salzano, Maria Luiza Petzl-Erler, Maria Claudia Nascimento, Philippe Mayaud, Jaila Dias Borges, Claudio S. Pannuti

**Affiliations:** Universidade de São Paulo, São Paulo, Brazil (V.A.U.F. Souza, C.S. Pannuti, M.C. Nascimento, J.D. Borges); Universidade Federal do Rio Grande do Sul, Porto Alegre, Brazil (F.M. Salzano); Universidade Federal do Paraná, Curitiba, Brazil (M.L. Petzl-Erler); London School of Hygiene and Tropical Medicine, London, UK (M.C. Nascimento, P. Mayaud).

**Keywords:** Viruses, herpesvirus, seroprevalence, South America, Native Americans, HHV-8, Amazonia, dispatch

## Abstract

To determine the epidemiology of human herpesvirus type 8 (HHV-8) among non-Amazonian native populations, we conducted a cross-sectional study in Brazil, Bolivia, and Paraquay. Our data show striking ethnic and geographic variations in the distribution of HHV-8 seroprevalences in Amazonian (77%) and non-Amazonian native populations (range 0%–83%).

Human herpesvirus type 8 (HHV-8) is the etiologic agent of all forms of Kaposi sarcoma, primary effusion lymphoma, and certain lymphoproliferative diseases. HHV-8 seroprevalence is low (<5%) in northern Europe and North America, where HIV-seropositive homosexual men represent the highest risk group. HHV-8 infection is endemic in eastern and central Africa, with seroprevalences >50% in some adult populations (*1*). However, the highest HHV-8 seroprevalences worldwide (>80% in adults) have been reported in Native Americans from the Amazon region of Brazil (*2*–*5*), French Guiana (*6*), and Ecuador (*7*). In HHV-8–endemic areas in Africa and the Amazon, the sharp and linear increases of HHV-8 seroprevalence in children before puberty, with only modest increases later in life (*3*,*5*), and the association of HHV-8 seropositivity in children with having at least 1 first-degree relative who is seropositive (*8*) suggest nonsexual transmission of HHV-8 within families, probably through saliva (*9*). Indeed, this hypothesis is supported by the finding that, in Native Americans, nearly one fourth of HHV-8–seropositive persons, and <40% of children, shed HHV-8 DNA in their saliva (*5*). A particular feature of the epidemiology of HHV-8 in Native Americans is that infection is caused by a recently discovered distinct and unique HHV-8 strain (subtype E) (*2*,*5*,*7*). Previous research has also shown that HHV-8 seroprevalence is 10-fold lower in nonnative populations living in similar conditions in adjacent remote geographic areas, which suggests that HHV-8 infection in Native Americans may be associated with specific risk factors or behaviors but not with environmental factors (*5*). Little is known, however, of the epidemiology of HHV-8 among other Native American populations. We conducted a cross-sectional study to investigate whether HHV-8 was also endemic among Native American populations living outside Amazonia.

## The Study

Serum samples from the Wai Wai, a Native American tribe living in the Mapuera village in the area of the Trombetas, a tributary of the Amazon River, in Pará State, Brazil, were collected during May–June 2007. In addition, we obtained archived serum samples (collected during studies conducted during1972–1988 and frozen at –20°C) from 5 non-Amazonian Native American populations (*10*–*13*). The following non-Amazonian ethnic groups were evaluated: 1) the Xikrin, Gorotire, and Kuben-Kran-Kegn, who live in a transition region between the Amazonian forest and the savannah of central Brazil; 2) the Krahó, from a savannah region of central Brazil; 3) the Ayoreo, from 3 different localities of the Chaco region of Bolivia and Paraguay; 4) the Kaingang, from Ivaí and Rio das Cobras in southern Brazil; and 5) the Guarani, from Rio das Cobras, in southern Brazil (Figure). The genetic and serologic studies were approved by the Brazilian National Ethics Commission (CONEP Resolutions. 123/98 and 005/2003).

**Figure:**
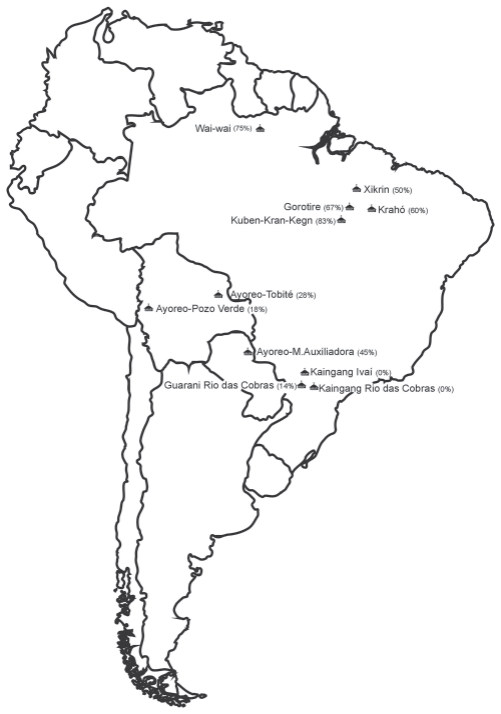
Locations and respective human herpesvirus type 8 seroprevalence rates (%) of Native American populations studied, South America.

Research aims and methods of the previous studies were diverse, but all enrolled participants (range 1–70 years of age) were enlisted without any previous selection from those who agreed to voluntarily participate in the study and convened at a specific place in the village to provide serum samples. Informed consent procedures were done individually, mostly verbally, with the assistance of local indigenous health agents. Consent for children <15 years of age was given by a parent or guardian (*10*–*13*). Antibodies to HHV-8 latency-associated nuclear (LANA) and lytic antigens were determined by immunofluorescence assays (IFA) at a 1:40 starting dilution, using the body cavity–based lymphoma (BCBL) 1 cell line. Punctuate nuclear staining in noninduced BCBL-1 cells was considered positive for LANA antibodies. The viral lytic cycle was induced by incubating BCBL-1 cells with 20 ng/mL of 12-O-tetradecanoylphorbol-13–acetate (TPA) (Sigma, St. Louis, MO, USA) for 96 hours. Entire cell fluorescence in ≈20% of TPA-treated cells was considered positive for antibodies to the lytic-phase antigens (*5*). Overall HHV-8 seropositivity was defined as positivity by any of these IFAs, but results are also presented by assay type. Chi-square statistics were used to estimate the associations between HHV-8 seropositivity and sex and age groups.

The prevalence of HHV-8 antibodies by any assay ranged from 0% to 83% in the various groups (Table 1). HHV-8 seroprevalence did not differ between men and boys (222/350, 63%) and women and girls (265/410, 64%) (p = 0.7). We found an overall significant trend for increasing prevalence with age (84/150, 56%) in children 0–12 years of age, 63% (282/450) in young adults 13–40 years of age, and 76% (121/160) in adults >40 years (p trend<0.001), although these results mostly reflected age seroprevalence patterns in the largest group (Wai Wai). The highest seroprevalences were observed in the populations living in the Amazon (77%) and in the transitional geographic area between the Amazon forest and the savannah of central Brazil (50%–83%). The Ayoreo from Bolivia and Paraguay had an intermediate seroprevalence (18%–45%), and the lowest seroprevalence was observed among Native Americans living in southern Brazil (Guarani and Caingang, 0%–14%), following a north-south gradient.

**Table 1:** Prevalence of HHV-8 antibodies in Amazonian and non-Amazonian Native American populations, by assay type*

Population, locality†	No. persons tested	No. (%) persons HHV-8 seropositive			
		By IFA-LANA or IFA-lytic	By IFA-LANA	By IFA-lytic	By IFA-LANA and IFA-lytic
Wai-Wai, Mapuera, Brazil	530	397 (75)	372 (70)	204 (38)	177 (33)
Xikrin, Caeteté, Brazil	30	15 (50)	13 (43)	6 (20)	4 (13)
Gorotire, PI Gorotire, Brazil	30	20 (67)	20 (67)	7 (23)	7 (23)
Kuben-Kran-Kegn, Nilo Peçanha, Brazil	30	25 (83)	25 (83)	4 (13)	4 (13)
Krahó, Cachoeira, Brazil	20	12 (60)	10 (50)	10 (50)	7 (35)
Ayoreo,Tobité, Bolivia	18	5 (28)	5 (28)	2 (11)	2 (11)
Ayoreo, Pozo Verde, Bolivia	17	3 (18)	2 (12)	1 (6)	0 (0)
Ayoreo, M Auxiliadora, Paraguay	20	9 (45)	9 (45)	7 (35)	7 (35)
Kaingang, Ivaí, Brazil	30	0	0	0	0
Kaingang, Rio das Cobras, Brazil	26	0	0	0	0
Guarani, Rio das Cobras, Brazil	29	4 (14)	4 (14)	2 (7)	2 (7)

*HHV-8, human herpesvirus type 8; IFA, immunofluorescence assay; LANA, latency-associated nuclear antigen. †Listed in a north–south gradient (Figure).

## Conclusions

Our data show striking geographic and ethnic variations in the distribution of HHV-8 seroprevalences among South American native populations. Previous reports have shown that most Amazonian Native American groups have high HHV-8 seroprevalence, but substantial between-group and sometimes within-group differences have been observed in previous studies (Table 2). These differences could be explained, to some extent, by the different techniques that have been used to detect HHV-8 antibodies. No clearly defined acceptable standard test exists to determine HHV-8 seropositivity, and different assays have shown different levels of accuracy, including intra-assay variations when the same test is performed in different laboratories (*14*). Therefore, comparing HHV-8 seroprevalence in different settings has limitations. In this study, the same assays (anti-LANA and anti-lytic IFAs) were performed on all samples from all populations in 1 laboratory, and major variations in the distribution of HHV-8 seroprevalence were observed. The low HHV-8 seroprevalence among the Guarani (14%) and its absence among 56 Kaingang persons, both populations living in southern Brazil, contrasts with the high seroprevalences in populations living in central Brazil and Amazonia.

**Table 2:** Previous human herpesvirus type 8 seroprevalence studies in Amazonian Native American populations*

Population†	No. positive/no. tested (%)	HHV-8 assay used‡	Reference
Wai Wai, Brazil	275/339 (81)	IFA-LANA, IFA-Lytic	(*5*)
Zoé, Brazil	18/49 (37)	EIA-ORF59/0RF65/K8.1	(*4*)
Emerillon, French Guiana	12/78 (15)	IFA-Lytic (ABI test)	(*6*)
Palikur, French Guiana	22/167 (13)	IFA-Lytic (ABI test)	(*6*)
Wayana, French Guiana	41/144 (28)	IFA-Lytic (ABI test)	(*6*)
Wayampi, French Guiana	93/302 (31)	IFA-Lytic (ABI test)	(*6*)
Wayampi, Brazil	85/123 (69)	IFA-LANA, IFA-Lytic	(*3*)
Wayampi, Brazil	28/33 (85)	IFA-LANA, EIA w-virus	(*2*)
Tiriyó, Brazil	381/664 (57)	IFA-LANA, IFA-Lytic	(*3*)
Tiriyó, Brazil	30/35 (86)	IFA-LANA, EIA w-virus	(*2*)
Tiriyó, Brazil	24/56 (43)	EIA-ORF59/0RF65/K8.1	(*4*)
Huaroni, Ecuador	38/38 (100)	IFA-LANA, EIA-K8.1, EIA-ORF73	(*7*)
Siona, Ecuador	10/41 (24)	IFA-LANA, EIA-K8.1, EIA-ORF73	(*7*)
Apalaí, Brazil	8/44 (18)	IFA-LANA, EIA w-virus	(*2*)
Arara-Laranja, Brazil	18/92 (20)	EIA-ORF59/0RF65/K8.1	(*4*)
Arara-Laranjal, Brazil	0/11 (0)	IFA-LANA, EIA w-virus	(*2*)
Kararao, Brazil	6/24 (25)	EIA-ORF59/0RF65/K8.1	(*4*)
Asurini-Koatinemo, Brazil	7/10 (70)	IFA-LANA, EIA w-virus	(*2*)
Munduruku, Brazil	36/40 (90)	IFA-LANA, EIA w-virus	(*2*)
Araweté, Brazil	12/17 (71)	IFA-LANA, EIA w-virus	(*2*)
Parakanã, Brazil	27/30 (90)	IFA-LANA, EIA w-virus	(*2*)
Xikrin, Brazil	20/34 (59)	IFA-LANA, EIA w-virus	(*2*)
Karitiana, Brazil	12/25 (48)	IFA-LANA, EIA w-virus	(*2*)
Suruí, Brazil	15/17 (88)	IFA-LANA, EIA w-virus	(*2*)
Mekranoti, Brazil	34/52 (65)	IFA-LANA, EIA w-virus	(*2*)
Cinta Larga, Brazil	1/14 (7)	IFA-LANA, EIA w-virus	(*2*)

*ABI, Advanced Biotechnologies Inc (Columbia, MD, USA); EIA, enzyme immunoassay; IFA, immunofluorescence assay; LANA, latency-associated nuclear antigen; ORF, open reading frame; w-virus, whole-virus assay. †Listed in north-south gradient (Figure). ‡Only series with >10 persons are presented.

Although this study lacks the power to assess statistically significant differences in HHV- 8 seroprevalences by geographic region, the variation in the distribution of HHV-8 is intriguing. Current consensus suggests that the Bering Strait land bridge was prehistorically the only entry point to the American landmass and that human migration occurred not earlier than 15,000 years ago, using the Pacific Coast route, with subsequent spread to the Amazon and the rest of South America. The presence of the Guarani and Kaingang at their present locations is thought to have occurred comparatively recently, ≈1,000–2,000 years ago (*15*). This history suggests that the differences in HHV-8 seroprevalence between these groups and others from the Amazon may be caused by more recent events.

Phylogenetic analysis of HHV-8 strains obtained from Native Americans living in different regions and from different groups could contribute to establishing the period when HHV-8 was introduced in Amazonian and non-Amazonian native populations. However, the use of archived serum samples in the present series precluded the exploration of genetic characteristics of HHV-8 strains in the non-Amazonian groups. Another limitation concerns the recruitment of study participants; thus, selection bias cannot be excluded. As previously reported (*5*,*7*), and, in contrast to other populations, all Native American groups exhibited a higher prevalence of antibodies against LANA than against lytic antigen, although the significance of this finding cannot be determined in this study. Larger prospective population-based studies and detailed historical, epidemiologic, and genetic investigations are needed to explore the substantial epidemiologic differences in HHV-8 infection found between Amazonian and non-Amazonian Native American populations.
